# Thioctamer: a novel thioctic acid–glatiramer acetate nanoconjugate expedites wound healing in diabetic rats

**DOI:** 10.1080/10717544.2022.2081382

**Published:** 2022-06-01

**Authors:** Nabil A. Alhakamy, Gamal A. Mohamed, Usama A. Fahmy, Basma G. Eid, Mohammed W. Al-Rabia, Amgad I. M. Khedr, Mohammed Z. Nasrullah, Sabrin R. M. Ibrahim, Ashraf B. Abdel-Naim, Osama A. A. Ahmed, Shadab Md

**Affiliations:** a Department of Pharmaceutics, Faculty of Pharmacy, King Abdulaziz University, Jeddah, Saudi Arabia; b Center of Excellence for Drug Research and Pharmaceutical Industries, King Abdulaziz University, Jeddah, Saudi Arabia; c Mohamed Saeed Tamer Chair for Pharmaceutical Industries, King Abdulaziz University, Jeddah, Saudi Arabia; d Department of Natural Products and Alternative Medicine, Faculty of Pharmacy, King Abdulaziz University, Jeddah, Saudi Arabia; e Department of Pharmacology and Toxicology, Faculty of Pharmacy, King Abdulaziz University, Jeddah, Saudi Arabia; f Department of Medical Microbiology and Parasitology, Faculty of Medicine, King Abdulaziz University, Jeddah, Saudi Arabia; g Department of Pharmacognosy, Faculty of Pharmacy, Port Said University, Port Said, Egypt; h Preparatory Year Program, Department of Chemistry, Batterjee Medical College, Jeddah, Saudi Arabia

**Keywords:** Alpha-lipoic acid, diabetic wounds, nanoparticles, HPMC hydrogel, TNF-α, IL-6

## Abstract

The current work aims to design thioctic acid (TA) and glatiramer acetate (GA) nanoconjugate (thioctamer) loaded hydrogel formula as well as evaluation of thioctamer preclinical efficacy in expediting wound healing in a rat model of the diabetic wound. Thioctamer was prepared by conjugation of GA and TA in a 1:1 molar ratio. Particle size, zeta potential, and thermodynamic stability of the prepared thioctamer were assessed. Thioctamer was loaded in hydroxypropyl methylcellulose-based hydrogel and *in vitro* release study was investigated. The ability of thioctamer to enhance the process of wound healing in diabetic rats was investigated by assessing wound contraction and immunohistochemical assessment of the inflammation markers IL-6 and TNF-α. The results demonstrated that thioctamer showed particle size of 137 ± 21.4 nm, polydispersity index (PDI) of 0.235, and positive zeta potential value of 7.43 ± 4.95 mV. On day 10 of making a skin excision, diabetic rat wounds administered thioctamer preparation showed almost complete healing (95.6 ± 8.6%). Meanwhile, % of wound contraction in animals treated with TA or GA groups exhibited values amounting to 56.5 ± 5.8% and 62.6 ± 7.1%, respectively. Histological investigation showed that the highest healing rate was noted in the thioctamer group animals, as the surface of the wound was nearly fully protected by regenerated epithelium with keratinization, with few inflammatory cells noticed. Thioctamer significantly (*p*<.05) inhibited IL-6 and TNF-α expression as compared with sections obtained from the negative control, TA, GA, or positive control group animals on day 14. The evidence of the ability of thioctamer to significantly expedite wound healing in the diabetic rats is presented.

## Introduction

Diabetes mellitus (DM) plagues many nations worldwide. The cause of this noninfectious chronic disease is pancreas failure to successfully come up with sufficient insulin or the body’s inability to effectively utilize insulin (Abdulaziz Al Dawish et al., 2016; Zimmet et al., 2016). It is expected that diabetes will become most prevalent in Middle East and North Africa (MENA) because of speedy economic development, urbanization as well as changes in how people live in the region (Alotaibi et al., 2017). It is estimated by International Diabetes Federation (IDF) that in 2019, 55 million adults between ages 20 and 79 have diabetes in the MENA Region. Further, the IDF estimates a rise in this figure to 108 million by 2045. Hyperglycemia affects about 11% of live births in the MENA region in pregnancy (IDF, 2019). Adults having diabetes in Saudi Arabia are 4,275,200. World Health Organization (WHO) reports that Saudi Arabia is second in list of countries where diabetes is prevalent in the Middle East region and is seventh worldwide (Abdulaziz Al Dawish et al., 2016). Diabetes brings about a number of acute complications like cardiovascular diseases, cerebrovascular diseases, renal disorders as well as obesity (Guo & DiPietro, 2010; Sailakshmi & Devi, 2016). Likewise, people with diabetes experience impairment when it comes to healing of serious wounds. Such individuals are susceptible to the development of acute non-healing diabetic foot ulcers (DFUs) (Guo & DiPietro, 2010). Right from the time of Hippocrates, wound healing has been deemed important (Farahpour et al., 2018). Wound healing is modulated by several mechanisms as well as physiological processes (Modarresi et al., 2019). The process is complex as it consists of a variety of interdependent stages, which includes hemostasis, inflammation, proliferation, as well as remodeling (Khezri et al., 2019).

When an individual is wounded, the initial phase of hemostasis starts instantly, and vascular constriction and fibrin clot is formed. There is production of pro-inflammatory cytokines and growth factors by the clot and surrounding wound tissue. After the control of bleeding, there is migration of inflammatory cells into the wound (chemotaxis) and facilitation of the inflammatory phase, involving sequential entry of neutrophils, macrophages, and lymphocytes. This is usually followed by the proliferative phase, overlapping with the inflammatory phase and it involves epithelial proliferation and movement above the provisional matrix in the wound, known as re-epithelialization. The final remodeling sets in and it is possible to happen for years. It is characterized by regression of numerous newly produced capillaries, toward returning the wound’s vascular density to normal (Guo & DiPietro, 2010).

**Figure 1.**
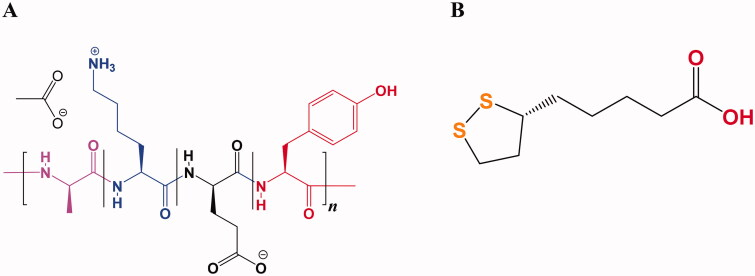
Chemical structure of GA (A) and TA (B).

Glatiramer acetate (GA, Figure 1(A)) is used for treating relapsing-remitting multiple sclerosis (RRMS) which is a central nervous system (CNS) inflammatory disease that damages the myelin sheath (Schellekens et al., 2011; Jürgens et al., 2016). This variability in molecular weight distribution influencing their properties regarding safety and efficacy. Therefore, GA also known as copolymer-1 (Cop-1) is certified nowadays in a number of countries around the world for multiple sclerosis (MS) treatment, it was officially approved in USA in 1996 by the Food and Drug Administration (FDA) and in 2001 by the European Medicines Agency (EMA) (Lalive et al., 2011; Alhakamy & Berkland, 2019). Along with its inherent complexities of structure, the average length of the polypeptide chains in GA is about 60 residues composed of four different amino acids at a defined molar ratio (l-alanine (0.427), l-lysine (0.338), l-glutamic acid (0.141), and l-tyrosine (0.095)) that create amphipathic property and positively charged. The average molecular weight ranges from 25 kDa to 200 kDa mostly 5–9 kDa. Therefore, Copaxone^®^ is considered as the first true nano-drug and can be regarded as protein where their molecule’s size in the colloidal suspension varies between 1.5 nm and 550 nm contributing to their biological activities that related with cytokines. The cationic residue lysine is the basic amino acid which is responsible for therapeutic effect, pre-clinical tests confirmed that when they showed inefficient finding of GA made with lacking of lysine (Schellekens et al., 2014; Alhakamy & Berkland, 2019; Prod’homme & Zamvil, 2019).

The mechanism of action of GA has long been an unclear concept despite of extensive investigations in both laboratory and clinical studies. It was originally recognized that GA therapy facilitated the proliferation of GA-reactive T-helper 2 and regulatory T cells and stimulated the neurotrophic factors to be released. The treatment by GA affects both adaptive and innate immune systems and it has become recognized that the main cellular targets for GA are antigen-presenting cells (APCs) like monocytes or dendritic cells that are accountable for the production of anti-inflammation T cells, resulting in beneficial therapeutic effects (Prod’homme & Zamvil, 2019). GA shows a significant binding to a complex major histo-compatibility (MHC) molecules and prohibits any response of T cells to the several myelin antigens. Also, it has been shown that GA plays a role as a T cell receptor antagonist for the 82–100 MBP epitope with continuously increased the anti-inflammatory cytokines secretion by inducing GA-specific regulatory CD8+ and CD4+ T cells and shifting TH1 to TH2 cells. GA-specific TH2 cells are capable of moving through the brain blood barrier and suppress *in situ* bystander of auto-aggressive TH1 T cells (Schrempf & Ziemssen, 2007).

Thioctic acid (alpha-lipoic acid, TA) is an oil and water soluble organo-sulfur metabolite that possesses two thiol groups (Figure 1(B)). It has been biosynthesized by animals, plants, and humans (Dragomanova et al., 2021). Additionally, it existed in supplements and foods and displayed diverse bio-activities such as antioxidant, immuno-modulatory, anti-cancer, anti-aging, anti-inflammatory, antiviral, and neuroprotective activities (Shay et al., 2009; Salehi et al., 2019; Anthony et al., 2021; Dragomanova et al., 2021). It improves utilization of glucose and boosts production of insulin, hence ameliorating hyperglycemia-induced oxidative damage (Salehi et al., 2019; Anthony et al., 2021; Dragomanova et al., 2021). Besides, it has been found to reduce and improve the diabetes-related disorders such as retinopathy and neuropathy, as well as autonomic neuropathy associated with diabetes (Gomes & Negrato, 2014; Dragomanova et al., 2021).

This work aims for thioctamer formulation (through the nanoconjugation of GA and TA) loaded hydrogel and to evaluate their preclinical efficacy in expediting wound healing in a rat model of diabetic wound.

## Materials and methods

Thioctic acid and hydroxypropyl methylcellulose (HPMC) were procured from Sigma-Aldrich Corp. (St. Louis, MO). Glatiramer acetate was purchased from NATCO Pharma Limited (NATCO House, Hyderabad, India). 3,3′-Diaminobenzidine (DAB) was procured from R&D Systems (Minneapolis, MN). All other chemicals and solvents used were of analytical grade.

### Preparation and characterization of thioctamer

Thioctamer was prepared by conjugation of GA and TA in a 1:1 molar ratios. The ratio selection of the two constituents was chosen based on the formerly reported work as well as the laboratory preliminary investigation (Alhakamy et al., 2021). TA was solvated in deionized water with the assistance of triethanolamine in a 1:1 molar ratio. GA was dissolved separately in deionized water and then mixed with TA aqueous solution with vortex for 60 s.

### Thioctamer zeta potential, particle size, and thermodynamic evaluation

The prepared thioctamer was assessed for zeta potential and particle size utilizing a Zetasizer Nano ZSP (Malvern Instruments Ltd., Malvern, UK). Thioctamer dispersion was appropriately diluted with deionized water before measurement. Zetasizer set parameters were laser wavelength 633 nm; scattering angle 173; temperature 25 °C; medium viscosity and refractive index were 0.8872 cP, and 1.33, respectively. For thermodynamic evaluation, thioctamer dispersion was prepared by dilution of 0.5 mL of the sample into 20 mL deionized water. Thioctamer dispersion was subjected to three freeze–thaw cycles at −20 °C for 12 h and +20 °C for 12 h (Badr-Eldin et al., 2021). The sample was then inspected for particle size utilizing the same instrument at the same temperature used for determination of particle size. The average of three determinations was used in the result calculations.

### Thioctamer loaded hydrogel preparation

HPMC was dispersed in distilled water at 1.5% w/v concentration then thioctamer was mixed with the HPMC solution that was kept stirring using magnetic stirrer at ambient temperature. The hydrogels were kept for 24 h to swell at 8 °C. Further, hydrogels of TA and GA were separately prepared by utilizing the same described method for thioctamer hydrogel preparation. The viscosity of prepared thioctamer hydrogels was evaluated using viscometer.

### GA release from thioctamer

The release study of the thioctamer formula was carried out using a Franz diffusion cell apparatus (MicroettePlus™; Hanson Research, Chatsworth, CA). Samples (equivalent to 0.5 mg GA content) were placed in the donor chamber; to pass through, a cutoff molecular weight of 12 kDa was placed on the dialysis bags used as a membrane to the donor chamber. Phosphate-buffered saline (pH 7.0) was used as a diffusion medium, and samples were withdrawn automatically at 0.5, 1, 2, 4, 8, 10, 12, and 24 h. GA diffused was measured by HPLC as previously reported (Parra Cervantes et al., 2020).

### *In vivo* study

#### Animals

Fifty 210–240 g male Wistar rats were provided by KAU’s Animal Facility (King Abdulaziz University, Jeddah, Saudi Arabia) that were maintained on a 12-h dark–light cycle and a 22 ± 2 °C (Badr-Eldin et al., 2021). The animal care procedures were certified by Faculty of Pharmacy’s Research Ethics Committee (PH-1443-09). Induction of diabetes in rats was carried out as formerly stated (Ahmed et al., 2014). Prior to the study, rats were intra-peritoneally injected with streptozotocin (50 mg/kg) for two weeks. Go Accu-Chek was utilized for assessing the level of fasting blood glucose (Roche, Mannheim, Germany). Moderate-diabetic rats with level of fasting blood glucose (200–300 mg/100 mL) were chosen for the experiment.

### Animal wounding and treatment

Rats were anesthetized by xylazine and ketamine (10 mg/kg and 100 mg/kg, respectively IP injection). The dorsal surface was shaved and sterilized using povidone-iodine solution. One centimeter diameter excision circle was made on the sterilized area, and the wounds were cleaned using sterile saline solution and dried with sterile pads. Subcutaneous injection of 2% solution of lidocaine hydrochloride having epinephrine (4.4 mg/kg) near to wound area for reducing pain (Labib et al., 2019). Five groups (six rats, each) of wounded-diabetic rats were organized according to the received treatment on the wounded area: group I received 1.5% w/v HPMC-based hydrogel (negative control); group II received HPMC hydrogel (1.5% w/v) preparation of 4.1 mg/g gel TA; group III received HPMC hydrogel preparation (1.5% w/v) of 12.5 mg/g gel GA; group IV received HPMC hydrogel (1.5% w/v) of 16.6 mg/g gel thioctamer complexes; group V received 0.5 g Mebo™ ointment (positive control). The treatments were daily applied topically for 14 days. The wounds were wrapped with sterile gauze dressings and exchanged daily. At day 0, 4, 10, and 14, wounds were photographed and assessed. Animals were sacrificed by beheading at the day 10 & 14 and the wound area skin was cut out. From each animal, a part of the obtained skin was retained in 10% neutral formalin, while the other part was preserved for further analyses at −80 °C.

### Measurement of wound

%Wound closure was estimated using the following equation, taking into account the wound diameter changes (Bae et al., 2005; Alhakamy et al., 2021):

(1) $$\text{\% of wound closure}=\frac{\text{wound diameter at day }0-\text{wound diameter on the last day}}{\text{wound diameter at day }0}\times 100\;$$

### Histological investigation

Wound tissues were preserved for 24 h in 10% neutral formalin, then dehydrated using ethanol serial concentrations, passing in xylene clearing agent, then introduced in paraffin (Labib et al., 2019). Tissues’ paraffin blocks were cut into 5 μm thickness that were rehydrated after dewaxing. Some sections were stained using eosin and hematoxylin (E&H), whereas the remaining were stained with Masson’s trichrome (MT) (Alturkistani et al., 2015; Labib et al., 2019). Histological investigation was carried out by a pathologist without beforehand knowing of groups’ treatment. On the basis of the abundance/degree of inflammatory cell infiltration, proliferation of fibroblast, deposition of collagen, angiogenesis, tissue granulation, and re-epithelialization scores were allocated in a range from − to +++.

### Immuno-histochemical estimation of TNF-α and IL-6 expression

Drying, deparaffinization, rehydration, and boiling of each tissue section in citrate buffer for 10 min at pH 6.0 were carried out. Tissue and Cell Staining Rabbit Kit having secondary antibody, blocking solution, and 3,3′-diaminobenzidine (DAB) from R&D Systems (Minneapolis, MN) were utilized. After that, the sections were incubated for 120 min in 5% bovine serum albumin followed by overnight incubation at 4 °C with anti-TGF-β1 or anti-VEGF-antibodies (1 μg/ml). After washing, slides were kept at RT for 60 min with the biotinylated secondary antibody and then were washed by PBS having 0.5% tween 20. Thereafter, addition of DAB and observation of color development were performed using microscope (1000 Nikon SMZ, Tokyo, Japan) equipped with a digital camera (DS‐Fi1Nikon, Tokyo, Japan) and ImageJ analysis software (ImageJ, 1.46a, NIH, Bethesda, MD) was utilized (Bucolo et al., 1999).

### Statistical analysis

Data are shown off as mean ± SD. One-way analysis of variance (ANOVA) followed by Tukey’s post hoc test was utilized for multiple comparisons. All analyses were done using version 8.0 GraphPad Prism software^®^ (San Diego, CA). Only two-tailed *p* values <.05 were regarded as statistically significant.

## Results

### Preparation and characterization of thioctamer

**Figure 2.**
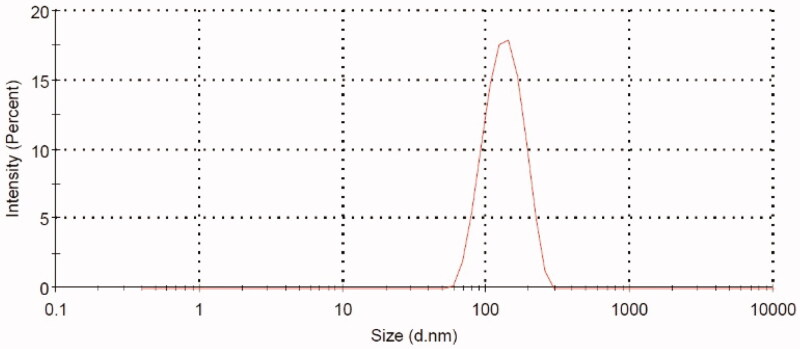
Thioctamer particle size as measured with the particle size analyzer.

**Figure 3.**
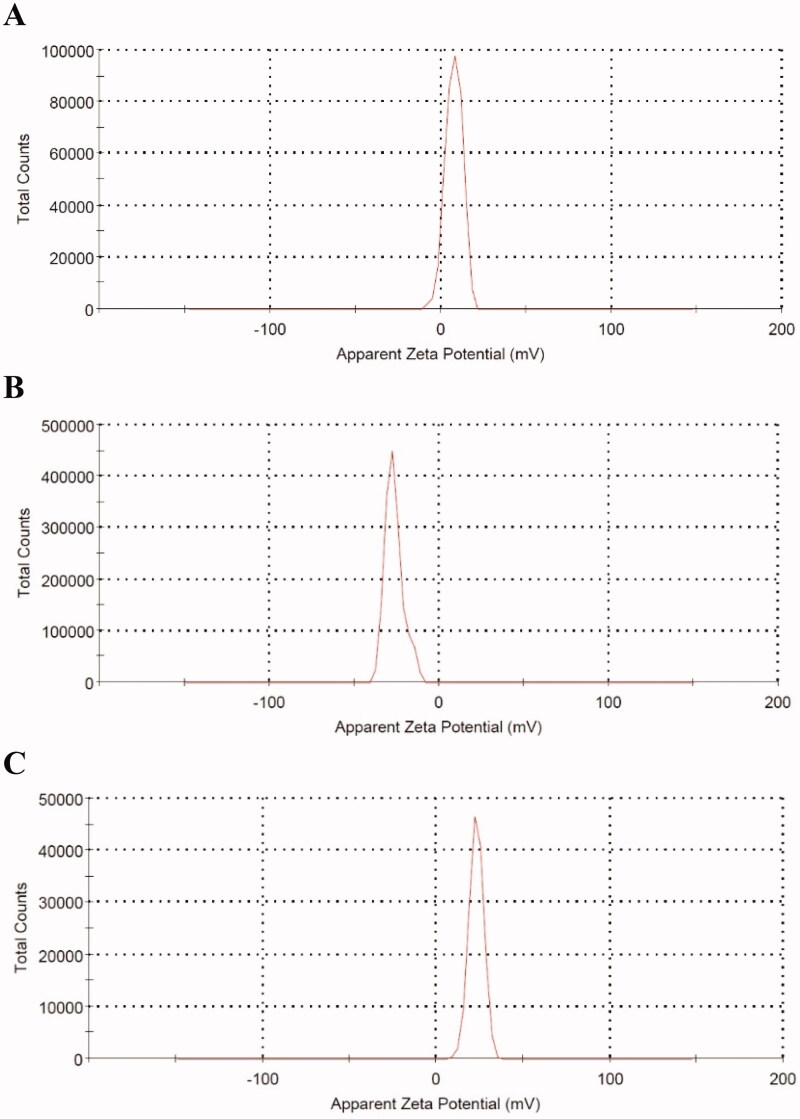
Zeta potential values of thioctamer (A), TA (B), and GA (C).

Thioctamer was prepared in GA:TA 1:1 molar ratio. The results demonstrated thioctamer particle size of 137 ± 21.4 nm (*Z*-average 166.4 nm) with polydispersity index (PDI) of 0.235 (Figure 2). Thioctamer showed a positive zeta potential value of 7.43 ± 4.95 mV (Figure 3(A)) when compared with the negative zeta potential value of TA of −26.6 ± 5.39 (Figure 3(B)) and the positive zeta potential value of GA 23 ± 4.2 mV (Figure 3(C)). These results revealed that thioctamer showed a net positive charge after the conjugation of TA (negatively charged) and GA (positively charged). The viscosity of thioctamer was found to be 2742 ± 3.4 cp. For thioctamer thermodynamic results, the findings (data not shown) indicate good thermodynamic stability of thioctamer with no significant differences in particle size after and before the three freeze–thaw cycles.

### GA release from thioctamer

**Figure 4.**
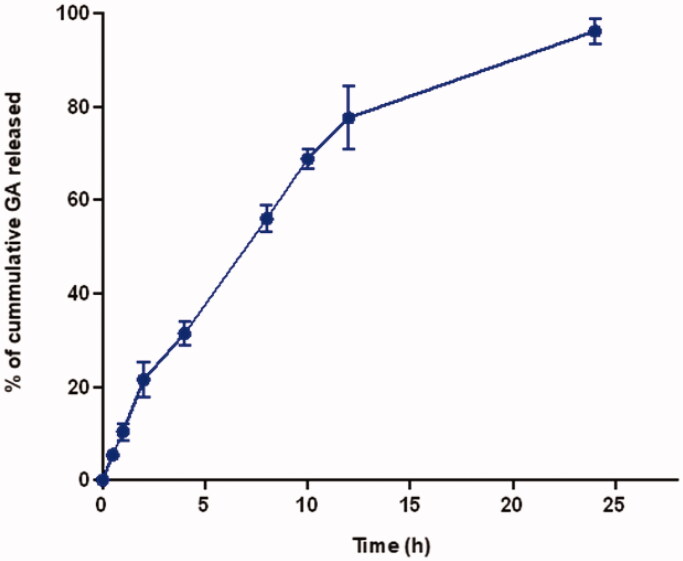
*In vitro* GA release from the thioctamer formula. Data represented as mean ± SD (*n* = 3).

As revealed in Figure 4, GA showed a sustained release profile over the 24 h study period. GA showed a 31.4 ± 2.6% cumulative amount released after 4 h and almost all the content (96.2 ± 2.7%) at 24 h.

### Assessment of wound healing

**Figure 5.**
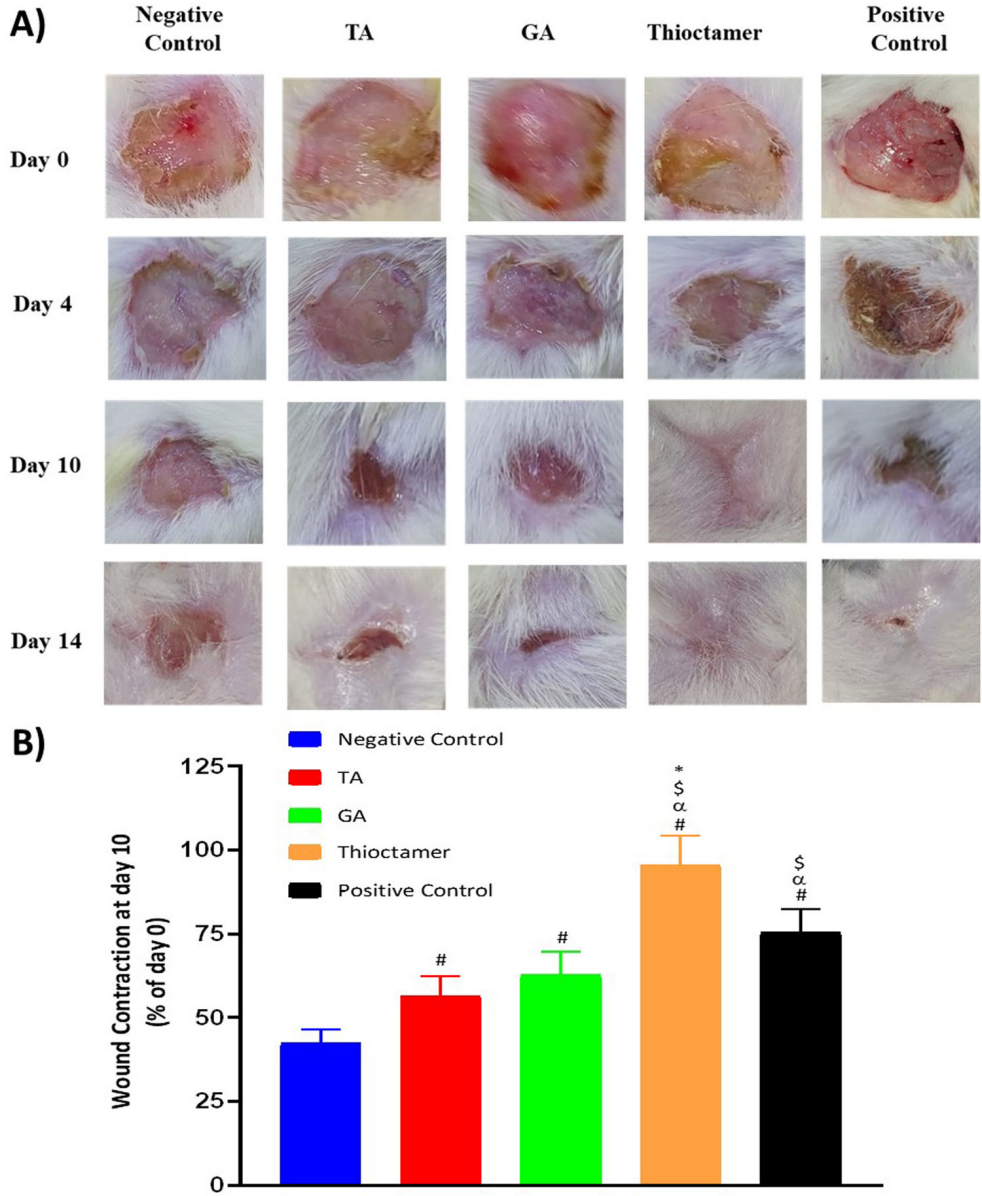
(A) Wound closure in diabetic rats at day 0, 4, 10, and 14 in the five experimental groups. (B) Wound contraction % at day 10. Data are shown as mean (*n* = 6)±SD. ^#^Significantly varied vs. negative control, ^α^significantly varied vs. TA, ^$^significantly varied vs. GA, and *significantly varied vs. positive control.

At day 10, thioctamer preparation applied to diabetic rat wounds showed almost entire healing (95.6 ± 8.6%). Meanwhile, % of wound contraction in animals treated with of TA or GA groups exhibited values amounting to 56.5 ± 5.8% and 62.6 ± 7.1%, respectively. It is noteworthy that thioctamer preparation significantly expedited wound healing in comparing to the positive control (Figure 5). This superior wound healing activity of thioctamer was still observed even at day 14.

### Histopathological investigation

**Figure 6.**
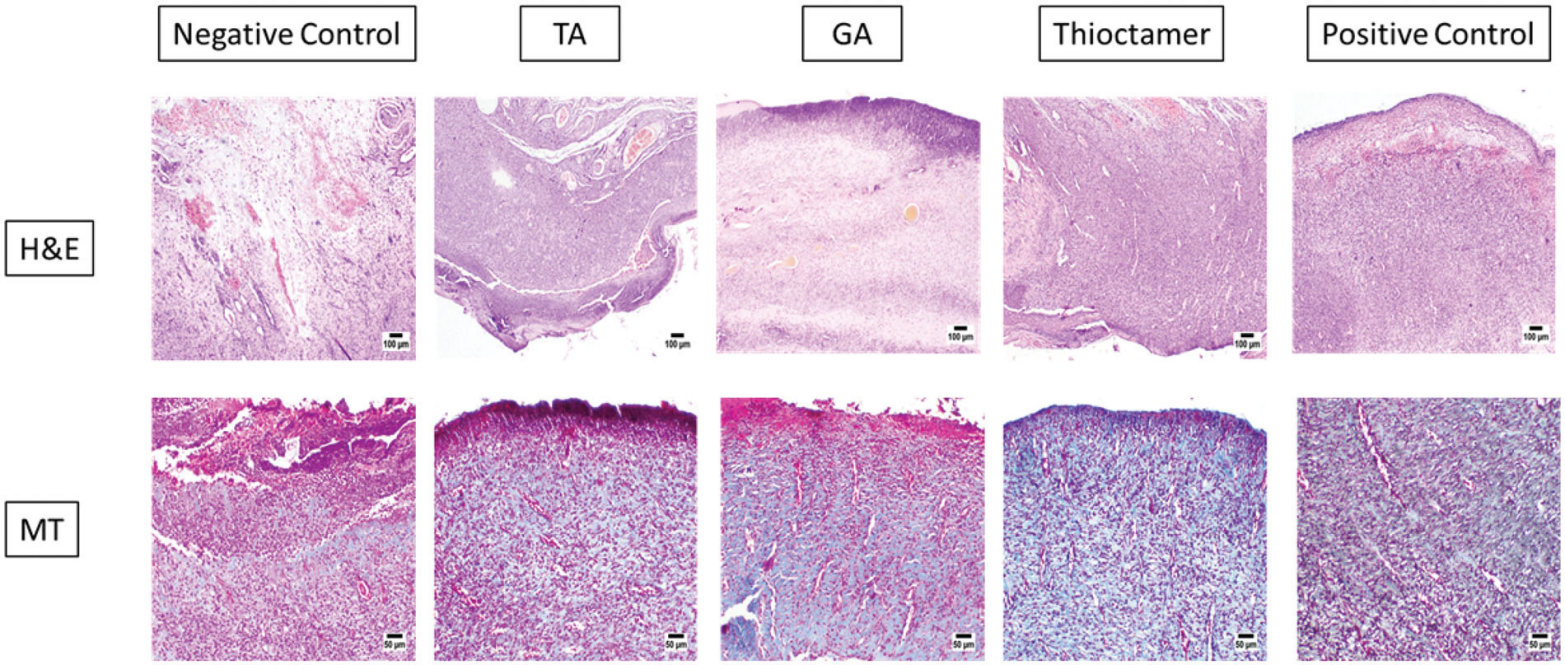
Histopathological effects of thioctamer loaded gel on wound healing on day 10. MT: Masson’s trichrome (scale bar = 50 µm); H&E: hematoxylin and eosin (scale bar = 100 µm).

The data from assessing the ability of thioctamer preparation to expedite wound healing activity were further asserted by histological investigation. Staining with hematoxylin and eosin or MT of wound tissues collected on day 10 revealed that the negative control group animals demonstrated delayed healing signs and of weak epidermal remodeling and re-epithelialization (Figure 6). The wound was occupied by severely inflamed granulation tissue with excessive edema and hemorrhages in the wound base. Transmigration of heavy neutrophilic infiltration was recognized in the wound covering that was accompanied by abundant eosinophilic, karyorrhectic debris, and necrotic crust. Tissues collected from TA or GA treated animals possessed to a certain extent moderate healing rate as re-epithelialization intensely expanded into the wound center area. The freshly produced epithelium was vacuolated with early keratinization. The utmost healing rate among animal groups was from the thioctamer group, as the surface of the wound was nearly fully enclosed by regenerated epithelium with keratinization and only little inflammatory cells were noticed. Mature organized tissue filled the wounds. The positive control group displayed better healing as inflammation was prohibited and epidermal remodeling was remarked (Figure 6). The histological characteristics are recorded and listed in Table 1.

**Table 1.** Histological estimation of day 10 wound healing after topical application of TA, GA, or thioctamer nanocomplex.

	IC	FP	CD	GT	Ang	RE
Negative control	++++	++	+	+	–	–
TA	+++	++	++	++	+	++
GA	+++	++	++	++	+	++
Thioctamer	+	++++	++++	++++	+++	++++
Positive control	++	++	+++	+++	++	++

FP: fibroblast proliferation; IC: inflammatory cell infiltration; GT: granulation tissue; CD: collagen deposition; RE: re-epithelialization; Ang: angio-genesis.

The greatest vascular alteration and infiltration of inflammatory cells were recorded in the first group (negative control), while improvement of angiogenesis and inflammation were noticed in the four other investigated animal groups. As evidently signified by the scores of histopathological lesions, progress in epidermal remodeling in positive control group as well as of TA, GA, or thioctamer groups was distinguished relative to the first group (negative control). The treatment with thioctamer presented the top score for angiogenesis, collagen deposition, and re-epithelialization comparing with the positive control group as well as other groups.

### Immunohistochemical assessment of IL-6 and TNF-α expression

**Figure 7.**
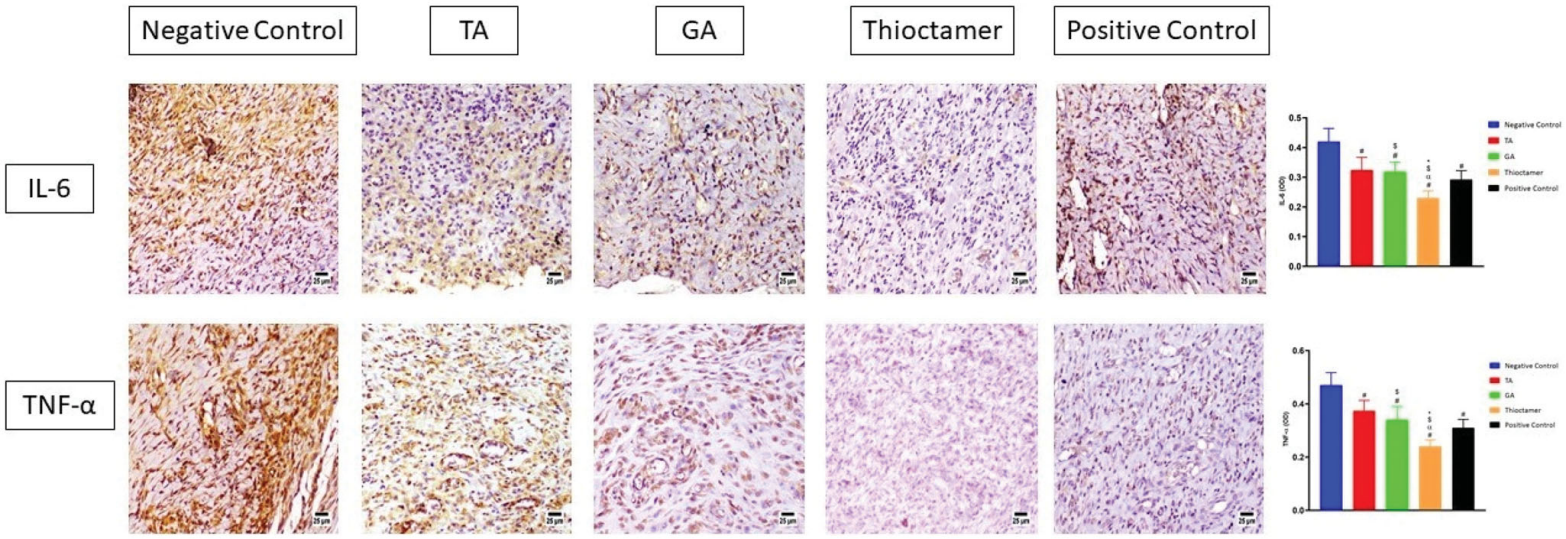
Effect of TA, GA, or thioctamer nanocomplex on TNF-α and IL-6 expression in diabetic rats wounded skin. Data are shown as mean ± SD (*n* = 6). ^#^Significantly varied vs. negative control, ^α^significantly varied vs. TA, ^$^significantly varied vs. GA, and *significantly varied vs. positive control.

The data in Figure 7 (upper panel) showed that treating of wounded skin tissues topically with TA, GA, or thioctamer remarkably prohibited IL-6 expression by 0.324 ± 0.043, 0.318 ± 0.033, and 0.23 ± 0.024, respectively, as compared to sections obtained from the first group (negative control) (0.42 ± 0.044). Similarly, TNF-α expression was significantly inhibited by TA, GA, and thioctamer by values amounting to 0.374 ± 0.039, 0.34 ± 0.05, and 0.241 ± 0.025, respectively, as compared to negative control (0.47 ± 0.048) (Figure 7, lower panel).

## Discussion

In uncontrolled diabetes, wound healing is a lengthy process that, if not treated properly, can result in serious consequences (Hong et al., 2014). It is well established that dysregulation of any of the wound healing processes can lead to the development of several persistent ulcers as well as an excessive scar formation (Efron & Moldawer, 2004; Hesketh et al., 2017). As a result of the above, it is obvious that innovative and effective pharmacological techniques are required to promote healing and restore the integrity of wounded tissue while evading the extreme repair associated with fibrotic skin.

The wound healing characteristics of thioctamer nanoconjugate were examined in diabetic rats to determine if TA and GA may interact synergistically to promote wound healing (Eming et al., 2007; Enoch & Leaper, 2008). The possible mechanism of conjugation is most probably attributed to the secondary types of bonding through possible charge attraction between the positively charged ammonium group of lysine amino acid of GA and the negatively charged carboxylate anion of lipoic acid. Thioctamer exhibited a size of <100 nm (98.7 ± 21.4 nm) and a 27.6 ± 3.2 mV zeta potential, which has optimal zeta potential for particle stability. Additionally, as previously demonstrated in earlier studies, the aforesaid particle size value enables maximal cellular absorption (Eming et al., 2007; Enoch & Leaper, 2008; Aya & Stern, 2014; Dunnill et al., 2015). The positively charged nanocarriers facilitate cellular entry (Bae et al., 2005). In this perspective, zeta potential is a critical measurement of colloidal stability, which affects various formulation characteristics as efficiency and performance.

Following production and characterization of thioctamer formulation that contains TA and GA in a 1:1 ratio, it was tested in an *in vivo* model (acute wound in diabetes rats). Generally, this innovative formulation outperformed the commercial available ointment, which served as positive control in this study. As shown in Figure 5, daily administration (topically for 14 days) of thioctamer loaded hydrogel on the generated wound accelerated the healing process, resulting in complete recovery, whereas positive control, TA, or GA treatments resulted in an incomplete recovery. Incidentally, it is worth noting that wound contraction after 14 days was statistically significant for all four treatments investigated in this work, even though thioctamer use was also significantly improved compared to other treatments in the study (Figure 5), implying pharmacological activity enhancement of TA and GA when conjugated in thioctamer. The wound healing properties of thioctamer were further examined histologically, demonstrating the formulation's potential to reduce inflammatory cell infiltration and improve parameters of tissue healing, which are predicted to accelerate the recovery (Figure 6 and Table 1).

IL-6 and TNF-α (inflammatory markers) have been demonstrated to be overexpressed in damaged tissues (Aya & Stern, 2010). Effective wound healing agent should inhibit IL-6 and TNF-α expression. On day 14, we determined the levels of IL-6 and TNF-α in the skin tissues of diabetic rats under a variety of experimental conditions. When diabetic mice were left untreated, all four therapies significantly reduced IL-6 and TNF-α levels (Figure 7). As demonstrated by wound contraction, thioctamer was the most efficacious, greatly surpassing all other regimens. The results of this section demonstrate that all four medications are effective at lowering IL-6 and TNF-α inflammatory mediators, demonstrating that TA or GA are less effective alone when compared with the combined thioctamer. The last stage of wound healing is characterized by remodeling, which is responsible for both the production of new epithelial cells and the development of scars (Wilgus et al., 2003; Desjardins-Park et al., 2018). This is a critical stage that is characterized by a delicate balance of degradation and synthesis that, in the best-case scenario, should result in the wound healing with minimal scarring. The findings of this investigation indicated that thioctamer treatment consistently had the greatest effect, always significantly greater than the other treatments, demonstrating the synergistic activity of TA and GA when formulated into nanoconjugate.

## Conclusions

The purpose of this study was to formulate a novel thioctamer loaded HPMC hydrogel in order to determine whether TA and GA compounds can interact synergistically and exert wound healing efficacy in an *in vivo* model of diabetic rat acute wound. In comparison to all other testing circumstances, thioctamer demonstrated increased and hastened excision wound healing characteristics. Thioctamer’s improved activity can be related to the reduced inflammatory cell infiltration and improve parameters of tissue healing, and anti-inflammatory properties. We think that the thioctamer formulation may provide a potential pharmacological tool for the creation of more tailored and effective wound healing formula for *in vivo* application in diabetic acute wounds.

## Data Availability

The data presented in this study are available in the article.
